# Comparison Between 30-15 Intermittent Fitness Test and Multistage Field Test on Physiological Responses in Wheelchair Basketball Players

**DOI:** 10.3389/fphys.2015.00380

**Published:** 2015-12-16

**Authors:** Thierry Weissland, Arnaud Faupin, Benoit Borel, Pierre-Marie Leprêtre

**Affiliations:** ^1^Laboratoire de Recherche Adaptations Physiologiques à L'exercice et Réadaptation à L'effort, EA-3300, UFR-STAPS, Université de Picardie Jules VerneAmiens, France; ^2^Institut d'Ingénierie de la Santé, UFR de Médecine, Université de Picardie Jules VerneAmiens, France; ^3^Laboratoire Motricité Humaine Education Sport Santé, EA-6312, UFR-STAPS, Université de ToulonLa Garde, France; ^4^Laboratoire Motricité Humaine Education Sport Santé, EA-6312, Université Nice Sophia AntipolisNice, France; ^5^Laboratoire Handicap, Activité, Vieillissement, Autonomie, Environnement, EA-6310, Département STAPS, Université de LimogesLimoges, France

**Keywords:** wheelchair, basketball, field test, aerobic fitness, evaluation

## Abstract

The intermittent nature of wheelchair court sports suggests using a similar protocol to assess repeated shuttles and recovery abilities. This study aimed to compare performances, physiological responses and perceived rating exertion obtained from the continuous multistage field test (MFT) and the 30-15 intermittent field test (30-15_IFT_). Eighteen trained wheelchair basketball players (WBP) (WBP: 32.0 ± 5.7 y, IWBF classification: 2.9 ± 1.1 points) performed both incremental field tests in randomized order. Time to exhaustion, maximal rolling velocity (MRV), *V*O_2peak_ and the peak values of minute ventilation (*V*_Epeak_), respiratory frequency (RF) and heart rate (HR_peak_) were measured throughout both tests; peak and net blood lactate (Δ[Lact^−^] = peak–rest values) and perceived rating exertion (RPE) values at the end of each exercise. No significant difference in *V*O_2peak_, *V*E_peak_, and RF was found between both tests. 30-15_IFT_ was shorter (12.4 ± 2.4 vs. 14.9 ± 5.1 min, *P* < 0.05) but induced higher values of MRV and Δ[Lact^−^] compared to MFT (14.2 ± 1.8 vs. 11.1 ± 1.9 km·h^−1^ and 8.3 ± 4.2 vs. 6.9 ± 3.3 mmol·L^−1^, *P* < 0.05). However, HR_peak_ and RPE values were higher during MFT than 30-15_IFT_(172.8 ± 14.0 vs. 166.8 ± 13.8 bpm and 15.3 ± 3.8 vs.13.8 ± 3.5, respectively, *P* < 0.05). The intermittent shuttles intercepted with rest period occurred during the 30-15_IFT_ could explain a greater anaerobic solicitation. The higher HR and overall RPE values measured at the end of MFT could be explained by its longer duration and a continuous load stress compared to 30-15_IFT_. In conclusion, 30-15_IFT_ has some advantages over MFT for assess in addition physical fitness and technical performance in WBP.

## Introduction

Wheelchair basketball (WB) attracts many persons with different physical impairment and has great success at the Paralympic Games since 1960. WB has been described as intermittent aerobic-based sport scattered with short anaerobic bouts (Coutts, 1992; Bloxham et al., 2001). In their game analysis, Sporner et al. (2009) reported that the wheelchair basketball players (WBP) on average traveled 2679 ± 1103 m cut off by 239.8 ± 60.6 stops and starts during a match. Wheeling tasks including sprint, endurance, and slalom were strongly correlated with aerobic fitness in WBP (Hutzler, 1993; Vanlandewijck et al., 1999). WBP presented larger cardiac dimensions, greater power output and peak oxygen uptake (*V*O_2peak_) values compared to untrained counterparts (Huonker et al., 1998; Schmid et al., 1998). Thus, maximal oxygen uptake was correlated to functional capacity and competition level in WBP (Schmid et al., 1998; De Lira et al., 2010).

Cardiorespiratory adaptation to exercise provided valuable information on training status. *V*O_2peak_ is generally assessed in laboratory condition during graded exercise performed on a wheelchair rolling on a motor driven treadmill and on an arm cycle ergometer. However, low correlations are obtained between peak cardiorespiratory values measured while pushing on the wheelchair and those measured with arm cranking on ergometer or in selected wheeling task (Hutzler, 1993; Rotstein et al., 1994). Standardized laboratory protocol tests can also provide higher *V*O_2peak_ reached at the end of test compared to field tests (Cunningham et al., 2000; Goosey-Tolfrey and Tolfrey, 2008). However, laboratory conditions did not take into account the natural environment (floor surface, specific wheelchair equipment) and not relate specific skills at the environment and ability to maneuver the wheelchair (Bernardi et al., 2010; Molik et al., 2010; De Groot et al., 2012; Goosey-Tolfrey and Leicht, 2013). Several authors adapted continuous (Vinet et al., 1996; Vanderthommen et al., 2002; Bernardi et al., 2010) and shuttle (Vanlandewijck et al., 1999; Cunningham et al., 2000; Goosey-Tolfrey and Tolfrey, 2008) tests for able-bodied players to assess aerobic fitness and predict the *V*O_2peak_ of disabled players. To assess agility, sprint recovery and endurance characteristics of WBP, Yanci et al. (2015) and Gil et al. (2015) also proposed a modified Yo-Yo intermittent recovery test (10-m instead of 20-m shuttle run). Yanci et al. (2015) showed a good test— retest reliability (ICC = 0.74–0.94; CV: ranged from 2.6 to 7.2%).

Buchheit (2008) developed for able-bodied athletes the 30-15 Intermittent Field Test (30-15_IFT_), which aims to evaluate the maximal aerobic velocity in court sport players and acute responses to high-intensity intermittent shuttle-runs. The main interests of this test is the final speed reached at the end of the test which is well suited for training prescription and the rest time is longer than the Yo-Yo intermittent recovery test and more representative of defensive phase of WP (Buchheit and Rabbani, 2014). Nevertheless, wheelchair sports are distinct from those able-bodied due to functional impairment of the disabled and the displacement imposed by wheelchair (Goosey-Tolfrey and Leicht, 2013).

Previously, an incremental multistage field test (MFT) specific for disabled body wheelchair subjects was validated (Vanderthommen et al., 2002). It was observed that a slightly MFT adaptation—as alternating right and left turns vs. single direction—increase *V*O_2peak_ and peak minute ventilation (*V*_Epeak_) responses without any significant differences in perceived exertion and maximal rolling velocity (MRV) reached at the end of the test (Weissland et al., 2015). These adjustments have no correspondence with the intermittent nature and the metabolic and cardio-respiratory responses induced by pivots, sprints and dribbles requested in WBP. Moreover, it has been observed, in able-bodied team sport players, that higher peak velocity were reached with a shorter time to exhaustion in intermittent shuttle vs. continuous running tests, with no significant difference in peak values of heart rate (HR_peak_) and blood lactate (Carminatti et al., 2013).

Hence, the aim of the study was (i) to assess the aerobic fitness derived from an able-bodied intermittent field test in WBP (Buchheit, 2008) and (ii) to compare with a continuous and validated wheelchair field test. This study aimed to examine the end-test rolling velocity, the physiological responses and perceived exertion obtained from the continuous MFT and with the 30-15 intermittent field test (30-15_IFT_).

## Methods

### Subjects

Eighteen national WBP were recruited and all were engaged in national WB competitions every week, with several training sessions per week. Skinfolds thickness at four sites (triceps, subscapular, suprailiac, and abdominal) was measured using a Harpenden caliper. A summary of their characteristics, pathology and international classification (International Wheelchair Basketball Federation Web site, 2009) is presented in Table 1. For both tests, all players always used their own wheelchair. Before each test, the tire pressure was checked (Sawatzky et al., 2005). All procedures were conducted in accordance with approval of the “Fédération Française Handisport” medical committee, and in accordance with the Helsinki Declaration. All participants are fully informed of any risk giving and provided written informed consent.

**Table 1:** **Individual Wheelchair basketball players' characteristics (gender, age, disability, sum of four skinfolds) according to International Wheelchair Basketball Federation classification (IWBF)**.

**Player**	**Sex**	**Age (years)**	**Disability**	**IWBF classification**	**ΣSK (mm)**
P1	M	29	Poliomyelitis	1.0	45.1
P2	F	28	Lower limb agenesis	1.0	76.2
P3	M	27	Spinal cord injury	1.5	52.2
P4	M	30	Spinal cord injury	2.0	42.9
P5	M	41	Spinal cord injury	2.0	27.2
P6	M	39	Spina bifida	2.5	33.7
P7	M	35	Hemiplegia	2.5	45.0
P8	M	36	Agenesis	3.0	44.9
P9	M	22	Larsen syndrome	3.0	30.8
P10	M	39	Spinal cord injury	3.0	39.1
P11	M	23	Spinal cord injury	3.0	37.9
P12	M	29	Cerebral palsy	3.0	44.1
P13	M	36	Spinal cord injury	4.0	48.3
P14	M	36	Spina bifida	4.0	54.5
P15	M	27	Cerebral palsy	4.0	23.1
P16	M	30	Above knee amputation	4.0	56.9
P17	F	38	Above knee amputation	4.5	34.1
P18	M	31	Orthopedic impairments	4.5	44.6
mean ± SD		32.0 ± 5.7		2.9 ± 1.1	43.5 ± 12.3

*ΣSK represents the sum of four skinfolds (biceps, triceps, subscapular, supra-iliac)*.

### Experimental design

Testing for this study was conducted during the competitive period, in the middle of the season. Both tests replaced technical and physical training sessions during a week between competitive matches. Training load was reduced on the day preceding each test, which was performed between 9:00 a.m. and 4:00 p.m. Each WBP performed both tests within 48 h in a randomized order, in the same indoor hall: (i) the MFT which is an incremental continuous test (Vanderthommen et al., 2002) and (ii) the 30-15 intermittent fitness test (30-15_IFT_) (Buchheit, 2008). Briefly, the MFT included wheeling around an octagon (15 × 15 m) at an initial speed of 6 km·h^−1^ during 1 min. Then, the speed increased by 0.37 km·h^−1^ every minute until exhaustion (Vanderthommen et al., 2002). The 30-15_IFT_ consisted of 40-m shuttle runs during 30-s with 15-s of passive recovery. The initial velocity was set at 6 km·h^−1^ (instead of 8 km·h^−1^ in the original protocol) for the first 30-s trial and was increased by 0.5 km·h^−1^ every 45-s (Buchheit, 2008). During the 15-s recovery period, the subjects rolled in the forward direction to join the closest line (at the middle or at one end of the area, depending on where they stopped) from where they started the next stage. No indication for the propulsion strategy was given for the two tests and WBP freely used their push rate and modality (synchronous and/or asynchronous).

All participants were instructed to complete as many stages as possible. The test ended when the participant could no longer be located within the turning zone (MFT) or consecutively to reach a 2-m zone around each line (30-15_IFT_) at beep signal despite verbal encouragement. The time to exhaustion (TTE) was the longer time maintaining to the speed imposed on the last stage during each respective test. MRV was the velocity at the end of test reached at the TTE.

All subjects were advised to keep the same meals between both tests and to refrain from smoking and caffeinated drinks during the 2 h prior to testing.

### Physiological and perceived responses measurements

The resting oxygen uptake (*V*O_2_), carbon dioxide production (*V*CO_2_), respiratory frequency (RF), and minute ventilation (*V*_E_) were measured breath-by-breath at rest and throughout both tests using Cosmed K4b^2^ or Metamax 3B portable spirometric systems. To reduce the duration of the test time and the turnover subjects, two portable measurement systems were used. A previous study showed a satisfactory comparison between the two measuring devices with able-bodied cyclists (Leprêtre et al., 2012). Participants always used the same analyzer for both tests to repeat the mistake device. The turbines flow meters (with a 3-L syringe) and analysers were calibrated before each test, according to the constructor instruction manuals using a two-point calibration (calibration gas O_2_ = 16% and CO_2_ = 5% against room air). Then we used the software of each device to automatically eliminate ectopic values and average the data every 5 s. Heart rate (HR) was continuously recorded beat-to-beat (Polar RS800, Polar Electro, Kempele, Finland) and averaged every 5 s.

Small capillary blood samples (0.5 μL) were collected from finger to assess basal lactate concentration. A sample of lactate at rest was taken upon arrival of the player and before warm-up, immediately after the test and 3 min after during the passive recovery period. Net blood lactate values (Δ[Lact^−^]) were calculated by the difference between the peak [Lact^−^] values and rest values. All blood samples were analyzed using a portable lactate analyzer (Lactate Pro, Arkray, Japan) calibrated before each test using a standard strip of provided by the manufacturer (Pyne et al., 2000).

Immediately after the end of both tests, participants individually rated their overall perceived exertion (RPE) using the Borg's 6–20 scale (Borg, 1990).

### Statistical analysis

Descriptive data are presented as mean and standard deviation (mean ± SD). Normality and homogeneity of the distribution were verified via Shapiro Wilks and Levene tests, respectively. Student's *t*-test was used to compare the resting and peak values measured during MFT and 30-15_IFT_. The determination of the Pearson correlation coefficients (R) were used to examine the relationship between TTE, MRV, *V*O2peak, and condition test. Absolute effect size (ES) and 95% confidence intervals of the differences (95% CI) were computed. An ES of 0.2 refers to a small effect, 0.5 a moderate effect, and 0.8 a large effect according to Cohen (Cohen, 1992). Agreements were sought by the Bland and Altman' method (Bland and Altman, 1986) between the peak values of *V*O_2_ and *V*_E_ between the both tests. In all statistical analyses, the (alpha) level of significance was set at *P* < 0.05.

## Results

Peak values of cardiorespiratory responses and performance measured during MFT and 30-15_IFT_ are shown in Table 2. Higher MRV values (14.2 ± 1.8 vs. 11.1 ± 1.9 km·h^−1^, *P* < 0.05, *ES* = 0.6) and shorter TTE (12.4 ± 2.4 vs. 14.9 ± 5.1 min, *P* < 0.05, *ES* = 0.3) were observed during 30-15_IFT_ compared to MFT. HR_peak_and RPE values were significantly lower during 30-15_IFT_ compared to MFT (166.8 ± 13.8 vs. 172.8 ± 14.0 bpm, *ES* = 0.4, and 13.8 ± 3.5 vs. 15.3 ± 3.8, *ES* = 0.5, *P* < 0.05, respectively). 30-15_IFT_ induced a higher Δ[Lact^−^] values compared to MFT (8.3 ± 4.2 vs. 6.9 ± 3.3 mmol·L^−1^, *P* < 0.05, *ES* = 0.4) without any significant difference between rest (*P* = 0.88) and peak [Lact^−^] values (9.8 ± 4.4 mmol·L^−1^ vs. 8.5 ± 3.1, *P* = 0.2, *ES* = 0.3). No significant difference was found for *V*O_2peak_, *V*_Epeak_, and RF peak values between both tests.

**Table 2:** **Peak values and 95% confidence interval (CI) measured during the MFT and the 30-15_IFT_, Mean ± SD**.

	**TTE min:s**	**MRV km.h^−1^**	**RF b^.^min^−1^**	***V*_Epeak_ L^.^min^−1^**	***V*O_2peak_ mL^.^min^−1.^kg^−1^**	**HR_peak_ bpm**	**peak [Lact^−^] mmol^.^L^−1^**	**Δ[Lact^−^] mmol^.^L^−1^**	**RPE**
MFT	14:53 ± 5:04	11.1 ± 1.9	49.9 ± 11.4	87.0 ± 22.8	33.0 ± 7.5	172.8 ± 14.0	8.5 ± 3.1	6.9 ± 3.3	15.3 ± 3.8
CI	12:32–17:13	10.3–12.0	44.7–55.2	76.5–97.6	29.5–36.5	166.3–179.2	7.1–9.9	5.4–8.4	13.5–17
30-15_IFT_	12:25 ± 2:21^*^^γ^	14.2 ± 1.8^*^^θ^	48.4 ± 12.8^θ^	84.4 ± 20.1	33.3 ± 7	166.8 ± 13.8^*^^θ^	9.8 ± 4.4^2^	8.3 ± 4.2^*^^θ^	13.5 ± 3.5^*^^θ^
CI	11:19–13:31	13.4–15.1	42.5–54.3	75.1–93.7	30.1–36.5	160.5–173.2	7.7–11.8	6.3–10.2	11.9–15.1

*TTE, indicates Time To Exhaustion; MRV, Maximal Rolling Velocity; RF, peak respiratory frequency; V_Epeak_, peak values of ventilation; VO_2peak,_ peak oxygen uptake; HR_peak_, peak heart rate; peak [La^−^], peak blood lactate value; Δ[La^−^], the difference between rest and maximal blood lactate values; RPE, the rating of perceived exertion*.

* Significantly different from MFT (P < 0.05);

θ moderate effect size;

γ *small effect size (Cohen, 1992)*.

**Figure 1:**
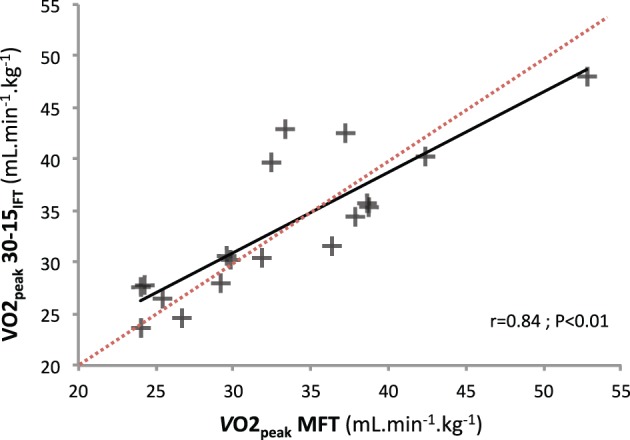
**Correlation between *V*O_2peak_ reached during MFT and 30-15_IFT_ tests (*r* = 0.54, *r*^2^ = 0.71, *P* < 0.01)**.

**Figure 2:**
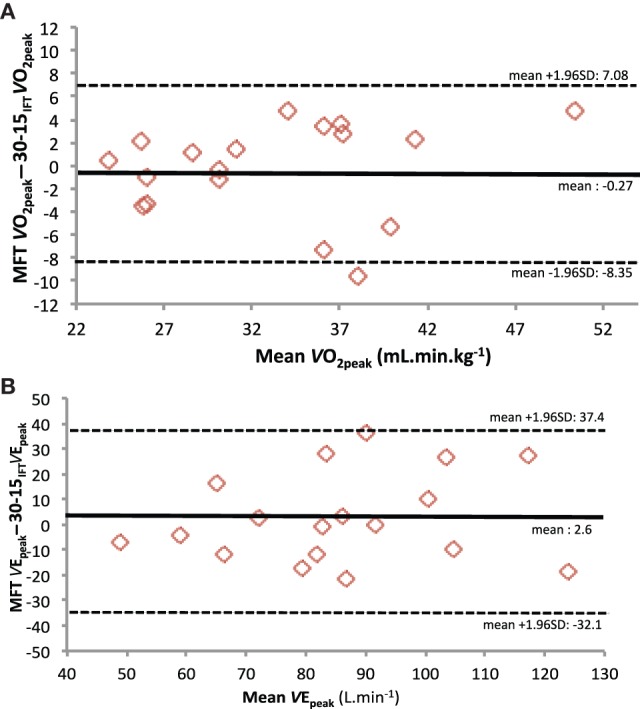
**Analysis of the individual difference by Bland-Altman method between MFT and 30-15_IFT_ test and (A) peak oxygen consumption (*V*O_2peak_) and (B) peak ventilation (*V*_Epeak_)**.

**Figure 3:**
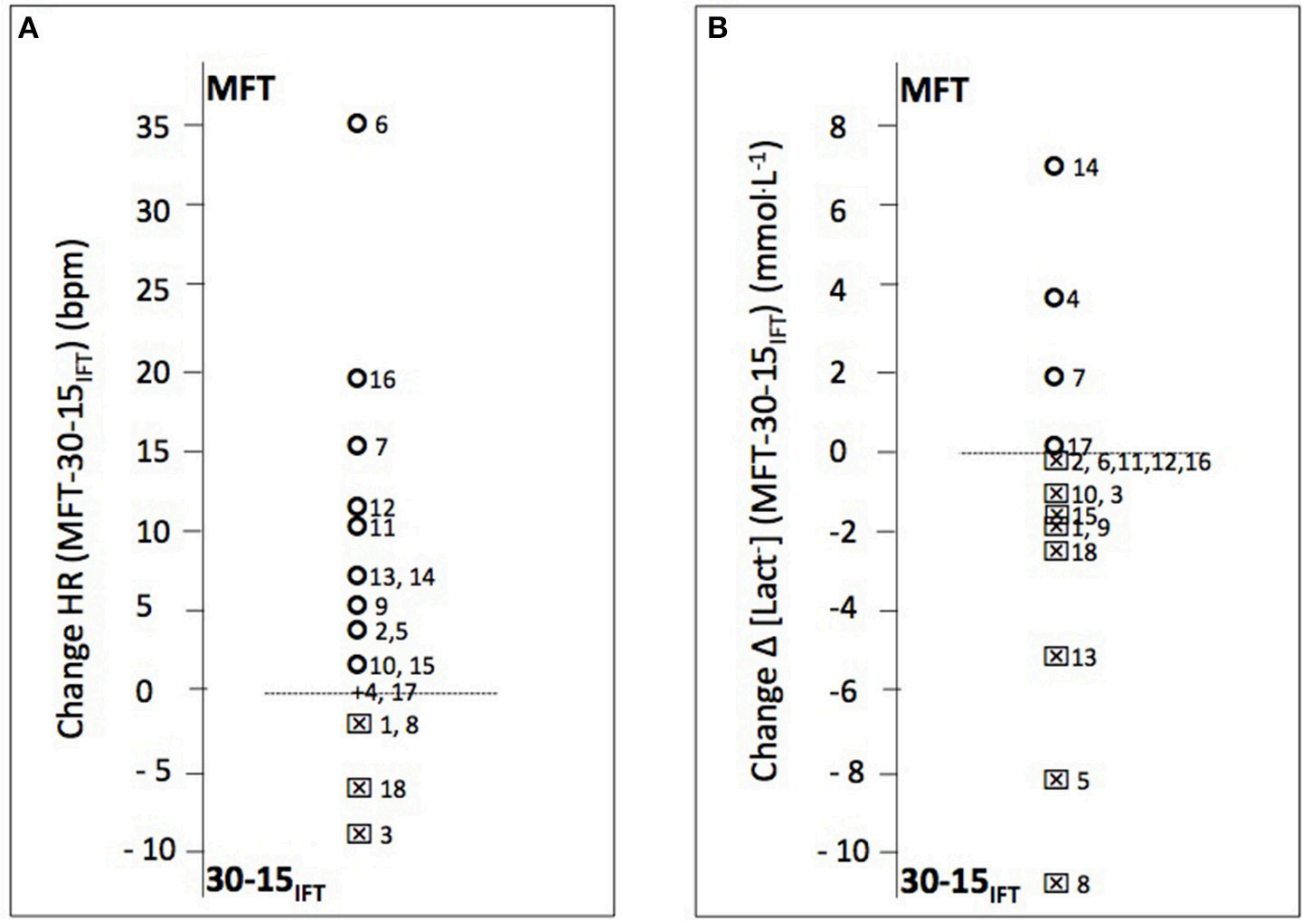
**Individual wheelchair basketball players difference in peak heart rate and blood lactate values between continuous multistage field test (MFT) and 30-15 intermittent field test (30-15_IFT_) (*n* = 18). (A)** Difference HR (bpm) and **(B)** Difference Δ[Lact^−^], respectively represented the difference in peak values of heart rate (bpm) and Δ blood lactate (peak-rest values [La^−^] mmol·L^−1^). A circle plots indicated a difference between MFT and 30-15_IFT_ values in favor of MFT; a square worth the 30-15_IFT_.

**Figure 4:**
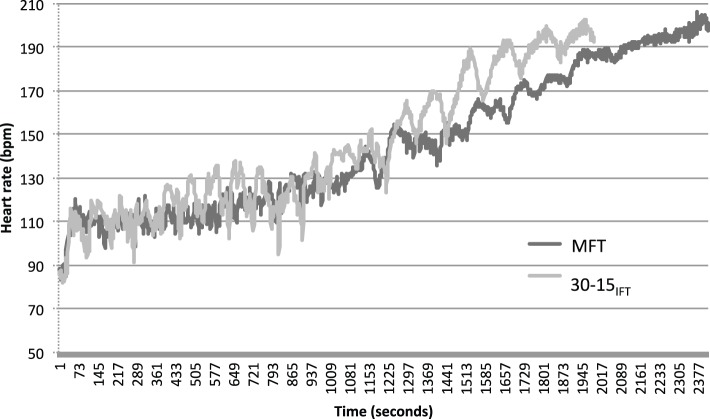
**Example data illustrating heart responses during 30-15_IFT_ and MFT for participant 12 (three point, IWBF classification)**.

A significant relationship for MRV (*r* = 0.57, *P* < 0.05) TTE (*r* = 0.64, *P* < 0.05), and *V*O2peak (*r* = 0.84, *P* < 0.01) was found between MFT and 30-15_IFT_(Figure 1). The Bland–Altman plots showed that, for *V*O_2peak_ and *V*_Epeak_ measurements, the bias ± random error was acceptable with an acceptable agreement between both tests (−0.27 ± 6.81 ml.min.kg^−1^; Figure 2A and 2.6 ± 34.8 L.min^−1^; Figure 2B, respectively). Differences between MFT and 30-15_IFT_ HR_peak_ and Δ[Lact^−^] per WBP were reported in Figure 3. Individual responses have reflected significant changes measured in HR (Figure 4) and Δ[Lact^−^] for 30-15_IFT_.

## Discussion

The aim of this study was to compare a modified able-bodied field intermittent test with a validated standardized wheelchair-users field test. The observed performances at 30-15_IFT_ were better with higher MRV associated with a shorter time to exhaustion (*P* < 0.05). However, no significant difference for peak oxygen uptake and ventilation values was noted between both tests (Table 2).

MFT is a validated field test to estimate *V*O_2peak_ for disabled body wheelchair subjects in indoor conditions (Vanderthommen et al., 2002). No significant difference was found for *V*O_2peak_ between MFT and 30-15_IFT_ and a significant relationship for *V*O_2peak_ were found between the both tests (*r* = 0.84, *P* < 0.01) (Figure 1). We used Bland-Altman plots to graphically display the variability of *V*O_2peak_ and *V*_Epeak_ variables (Figure 2). In each case, the systematic bias is close to zero and the 95% limits of agreements are acceptable. Thus, we can conclude that the 30-15_IFT_ is comparable to *V*O_2peak_ and *V*_Epeak_ encountered during the end of the test. Nevertheless, it would be necessary in the future to investigate the reliability and validity of the 30-15_IFT_ with a standardized test on a wheelchair ergometer in the laboratory. However, the only valid option to confirm whether a “true” *V*O_2max_ has been reached during both tests is currently in a verification phase (VER) (Leicht et al., 2013), absent in our protocol.

TTE to reach *V*O_2peak_ is shorter during 30-15_IFT_ than MFT (12.4 ± 2.4 vs. 14.9 ± 5.1 min, *P* < 0.05, *ES* = 0.3) and a significant correlation between both TTE tests was found (*r* = 0.64, *P* < 0.05). To reach *V*O_2max_, Buchfuhrer et al. (1983) recommended a time span of 10 ± 2 min for an incremental ramp protocol. This widely cited recommendation is applied for incremental protocols with able-bodied participants but also for upper body exercises and disabled subjects. With 16 untrained able-bodied men, Smith et al. (2006) observed, during an incremental arm crank ergometry, the effects of two ramp rates (12W·min^−1^ vs. 6W·min^−1^) on the attainment of peak physiological responses and power output (Smith et al., 2006). In this study, TTE was shorter for the 12W·min^−1^ protocol (within the range of 8–12 min) whereas, for the 6W·min^−1^ protocol, TTE extends to 15 ± 4 min. However, no significant difference was found for *V*O_2_, *V*_E_, and HR_peak_ between both protocols. In wheelchair athletes, Vinet et al. (1997) adjusted the velocity increment from a progressive treadmill test to be within the limits defined by Buchfuhrer et al. (8:50 ± 1:24 min). The Modified Yo-Yo intermittent recovery test (10 m shuttle run) provides higher TTE (16.96 ± 1.14 min), as reported by Yanci et al. (2015) in WBP. Considering the recommendations of time span (between 8 and 12 min) and given the absence of differences in the peak physiological responses, IFT_30−15_ would be more appropriate than MFT for the trained participants, due to the shorter TTE required for reaching *V*O_2peak_.

The difference in HR_peak_ measured at the end during both tests (166.8 ± 13.8 vs. 172.8 ± 14.0 bpm for 30-15_IFT_ and MFT, respectively, *ES* = 0.4, *P* < 0.05) can be explained by the intermittent nature of the 30-15_IFT_, which is based on the use of 15-s passive rest periods between each stage. HR responses represented as an example in Figure 4 for the 12 WBP clearly show the difference in HR evolution between the two tests. 30-15_IFT_ therefore allows a discontinuous load stress for WBP. 30-15_IFT_ does not elicit maximal HR and the maximal capabilities of the cardiovascular system while some other criteria for the attainment of a maximal exercise (like *V*O_2_ plateau, RER > 1.1, lactate concentration accumulates > 8 mmol.L^−1^) were achieved. A peripheral limitation may explain the submaximal values for HR_peak_. Vanlandewijck et al. (1999) has supported that shuttle test is not a direct measure of aerobic capacity but rather reflects ability and specific skills using the wheelchair. Indeed, Goosey-Tolfrey and Tolfrey (2008) showed that cardiorespiratory responses during a continuous shuttle multi-stage fitness test did not fully reflect those obtained during an exercise on arm crank ergometer. With WBP population, Yanci et al. (2015) reported greater HR_peak_ values (+4.7%) with a longest time (+36%) with Yo-Yo intermittent recovery test than the 30-15_IFT_. One other explanation can be the disruption of the autonomic control of the HR in three subjects with high spinal cord lesion, which would control the cardiovascular function during exercise and rest (West et al., 2014).

Peak RPE measured during MFT are consistent with the level of cardio-respiratory solicitations but RPE should be used cautiously for spinal cord injury athletes and differentiated for high and low lesion (Goosey-Tolfrey et al., 2010). Test duration and monotony of continuous displacement in MFT could increase the overall rate of perceived exertion during MFT in comparison with 30-15_IFT_ (15.3 ± 3.8 vs. 13.8 ± 3.5 *ES* = 0.5, *P* < 0.05). Turning in the same direction during MFT could induce premature tiredness and muscle fatigue in the upper limb of the external curve. This could be in relation to the great push power output and high arm frequency and the centrifugal force exerted on the wheelchair in the curve. With novice wheelchair users, Paulson et al. (2013) showed self-regulation of intermittent exercises based on the overall or peripheral perceptions. Dissociating muscular and respiratory RPE in order to analyze match load is a feasible method of quantification in monitoring the training of WBP (Iturricastillo et al., 2015). In our study, overall RPE did not provide information of the muscular load perceived by the succession of starts and changes of direction in the 30-15_IFT_'s protocol. An evaluation of peripheral RPE would certainly have given additional information between the two tests.

Higher MRV values were reached in a shorter time during 30-15_IFT_ compared to MFT (14.2 ± 1.8 vs. 11.1 ± 1.9 km·h^−1^, *P* < 0.05, *ES* = 0.6). 30-15_IFT_ adaptation by initially starting at 6 km·h^−1^ allowed to extend the standard protocol of 3-min, in order to have the same initial velocity between MFT and 30-15_IFT_. Despite this modification, 30-15_IFT_ induces shorter TTE. It is explained by the difference in less than 15-s and in addition to 0.13 km·h^−1^ per stage in detriment to MFT between the both tests. Also for these reasons, MRV attained higher values at 30-15_IFT_that MFT for similar *V*O_2peak_.

Higher 30-15_IFT_'s MRV could explain the higher peak blood lactate values. Smith et al. (2006) measured higher peak [Lact^−^] for 12 W·min^−1^ ramp protocol and had argued that the higher workload increment than 6 W·min^−1^ was linked with higher lactactes concentration. In this study, the workload during the graded exercise has an impact on the muscular component. Thus, compared with continuous octagonal line rolling in MFT, 30-15_IFT_ with direction changes and multiple acceleration phases could present a greater physiological load, as supported by relative blood lactate concentration and the extra energy expended. Δ[Lact^−^] 30-15_IFT_ values compared to MFT were higher (8.3 ± 4.2 vs. 6.9 ± 3.3 mmol·L^−1^, *P* < 0.05, *ES* = 0.4). The significant increase of velocity per 0.5 km·h^−1^ at IFT after rest period, added to direction changes, deceleration and acceleration phase generate significant muscular efforts and greater anaerobic solicitation. However, Yanci et al. (2015) reported, for the modified Yo-Yo protocol, lower peak [Lact^−^] values than our data (7.21 ± 2.4 mmol·L^−1^ vs. 9.8 ± 4.4 mmol·L^−1^). Intermittent field tests correspond to the nature of the court sport in WB. Comparing Yo-Yo and 30-15_IFT_ with young soccer players, Buchheit and Rabbani (2014) have noted a large correlation (*r* = 0.75) between both tests, with 30-15_IFT_ being more related to maximal sprinting speed and Yo-Yo being more associated with aerobic endurance. Bloxham et al. (2001) showed with the Canadian team that 28% of the WB playtime was spent at high anaerobic intensities and estimated 20% above the ventilatory threshold. But, 48.3% of playtime concerns recovery and low-speed replacement periods. Considering these aspects, 30-15_IFT_ could be closer to the WB conditions than the Yo-Yo intermittent test. Intermittent field testing would also have the advantage in determining the BLa threshold rather than ventilatory data collection (Leicht et al., 2014) with the sample blood lactate at each level.

Using able-bodied field tests to assess the physical condition of athletes with disabilities remains difficult and even imperfect (Goosey-Tolfrey and Leicht, 2013). First, pushing for inducing wheelchair rolling is not comparable to running. The amount of energy required for the inertia of the wheelchair are different, especially to start, to turn or to glide at half turn. Secondly, the initial rolling velocity is often inappropriate and the increment may be too important. In these conditions, shuttle protocols need a great technique or ability to maneuver the wheelchair and could limit the wheelchair novices and players with a low classification point (IWBF) that have more significant disabilities than the others. Shuttle tests could be more disadvantageous for athletes with greater disabilities than MFT in which the participants determine their preferred direction of rotation (Vanlandewijck et al., 1999). Functional asymmetry with a dominant side and contralateral side deficit in strength, imbalance had low impact for the physiological responses and MRV that are related between both tests (*r* = 0.57, *P* < 0.05). Heterogeneity of pathologies and residual functional capabilities represented in our study provide individual responses as shown in Figure 3.

Maneuvering a wheelchair during acceleration-deceleration phases, slide and half turns requires specific skills, considering individual muscular impairments and trunk imbalance. A moderate to high level of expertise of these techniques is essential for not stopping prematurely in 30-15_IFT_. It would be useful to compare our results during 30-15_IFT_ with other untrained or novice wheelchair users in order to determine if 30-15_IFT_ is also adaptable to various populations like MFT. Sprinting ability and wheelchair maneuverability are probably important predictors of performance in WBP (Vanlandewijck et al., 1999; Granados et al., 2015; Yanci et al., 2015). Thus, the 30-15_IFT_ has the advantage to assess, in addition to physical fitness, the technical performance to maintain wheeling velocity with succession of alternating turns. MFT was originally developed to assess aerobic fitness of the wheelchair users without a GTX laboratory protocol. MFT is validated and the *V*O_2peak_ extrapolation equation from MFT-score is reliable, repeatable and similar to *V*O_2peak_ measured (Vanderthommen et al., 2002; Weissland et al., 2015). However, MFT protocol is not representative of the WB nature while intermittent field tests are more similar but would need assessment to determine their level of reliability, validity and sensitivity.

As a take home message, the MFT test is more appropriate for the determination of maximal physiological capacities of WBP and the associated MRV can be used for the individualization of pre-season training programs. However, with a shorter time to exhaustion, 30-15_IFT_ is also really interesting and relevant for the evaluation of the WBP. This intermittent field test allows reaching *V*O_2peak_ with a higher contribution of the anaerobic metabolism, while also assessing and taking into account the specific technical characteristics of WB. The important differences for peak HR, [Lact^−^] and MRV values between both tests emphasize the importance of an adequate and relevant test selection, according to the parameter of interest.

## Conclusion

The 30-15 Intermittent Fitness Test induced a higher MRV with a greater blood lactate value but lower heart rate and perceived exertion compared to the original continuous MFT. Moreover, time of exhaustion is shorter for 30-15_IFT_ with similar peak oxygen uptake reached at the end of both test. Intermittent field test has some advantages over MFT for jointly assessing physical fitness and technical ability of WBP. It would be necessary in the future to investigate the reliability and validity from a standardized test on wheelchair ergometer in the laboratory.

### Conflict of interest statement

The authors declare that the research was conducted in the absence of any commercial or financial relationships that could be construed as a potential conflict of interest.
